# Supplementary material for: Diverse Populations of Staphylococcus pseudintermedius Colonize the Skin of Healthy Dogs
Source: Microbiol Spectr. 2023 Feb 14;11(2):e03393-22. doi: 10.1128/spectrum.03393-22 (PMC10100665; doi:10.1128/spectrum.03393-22)
Supplement: Supplemental file 2 — Supplemental material. Download spectrum.03393-22-s0002.pdf, PDF file, 11.4 MB [file spectrum.03393-22-s0002.pdf]

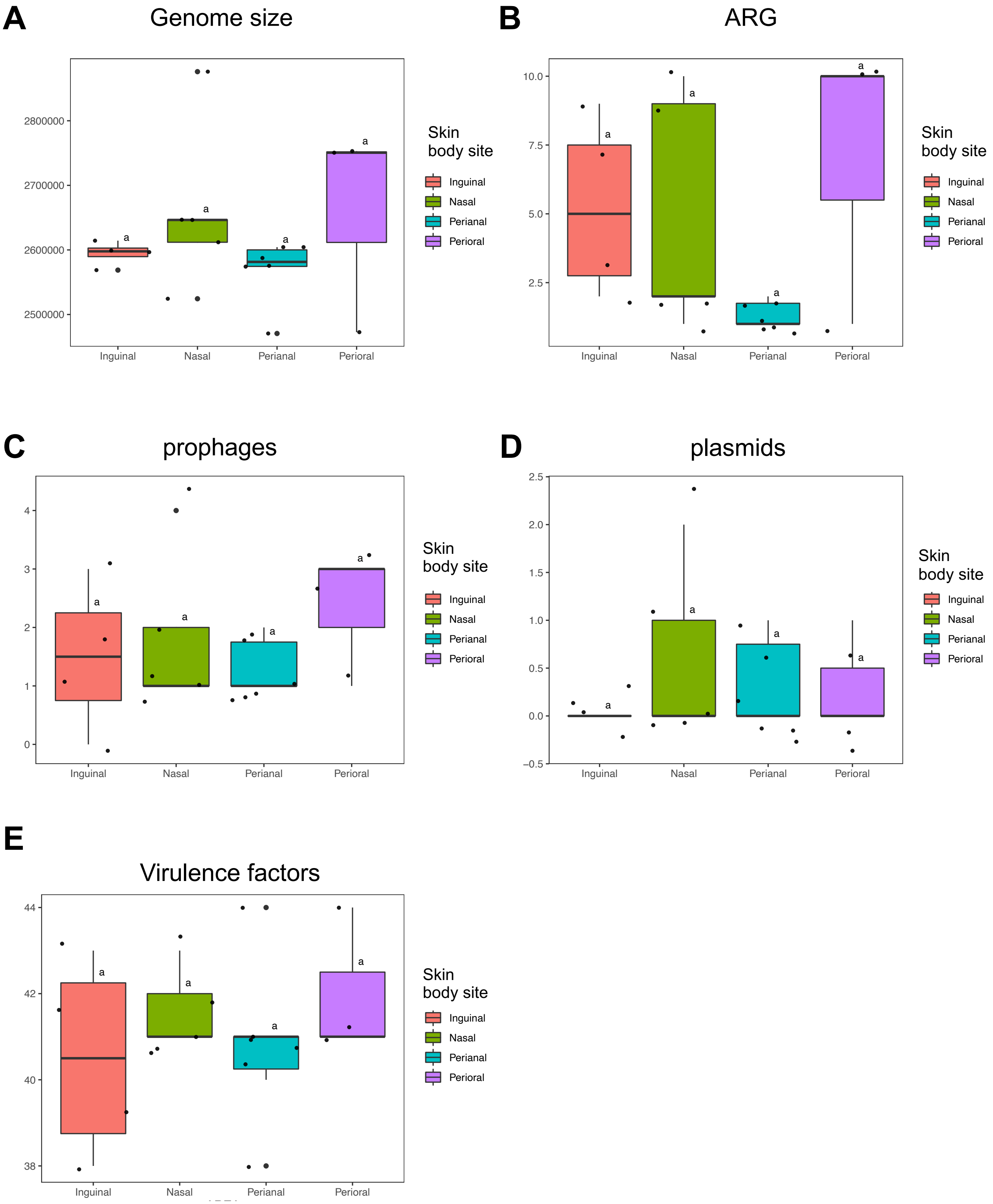

Supplementary Figure 1. No significant differences were detected between *S. pseudintermedius* genomes from different skin body sites.

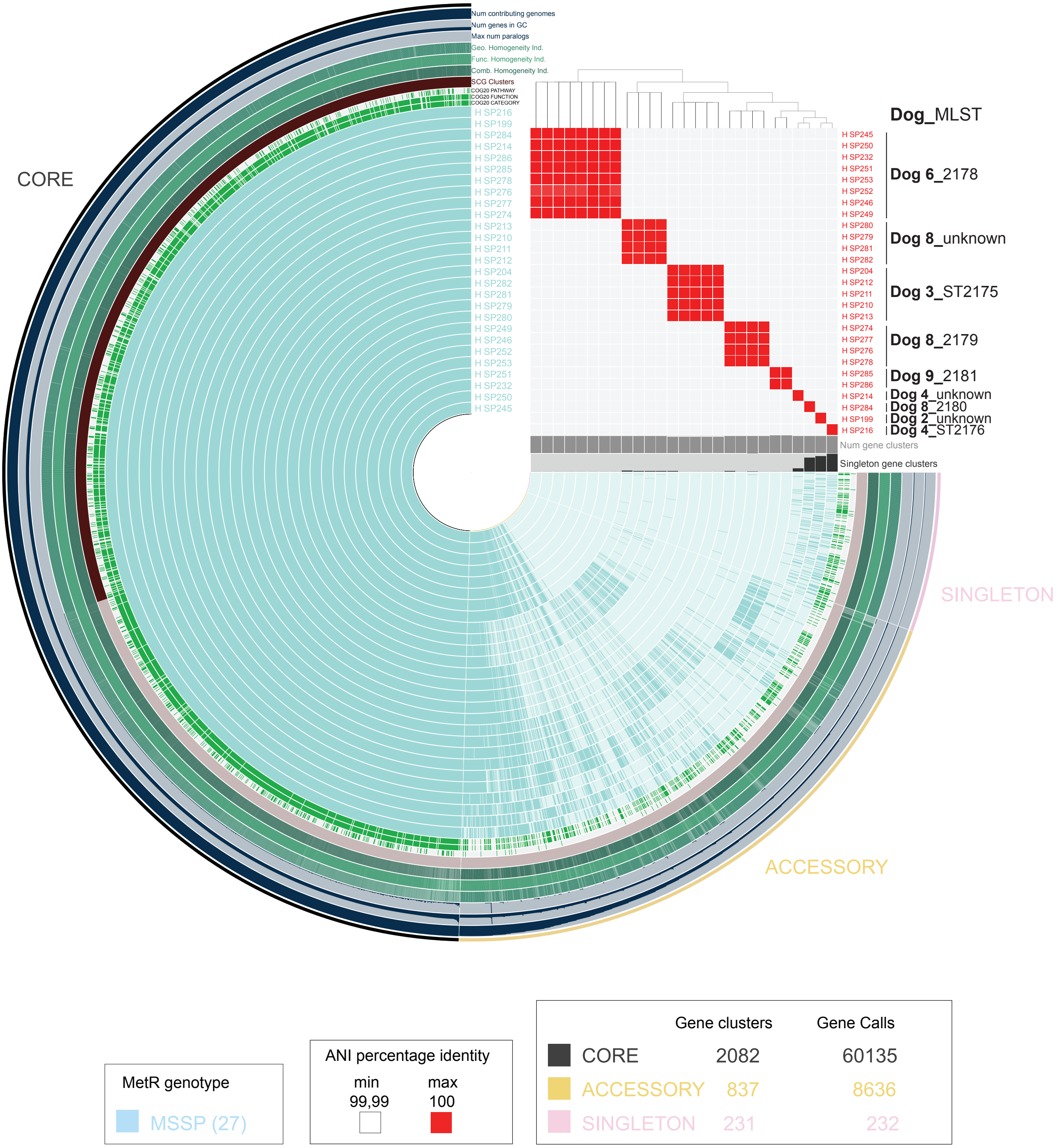

Supplementary Figure 2. Pangenome analyses of MSSP genotypes

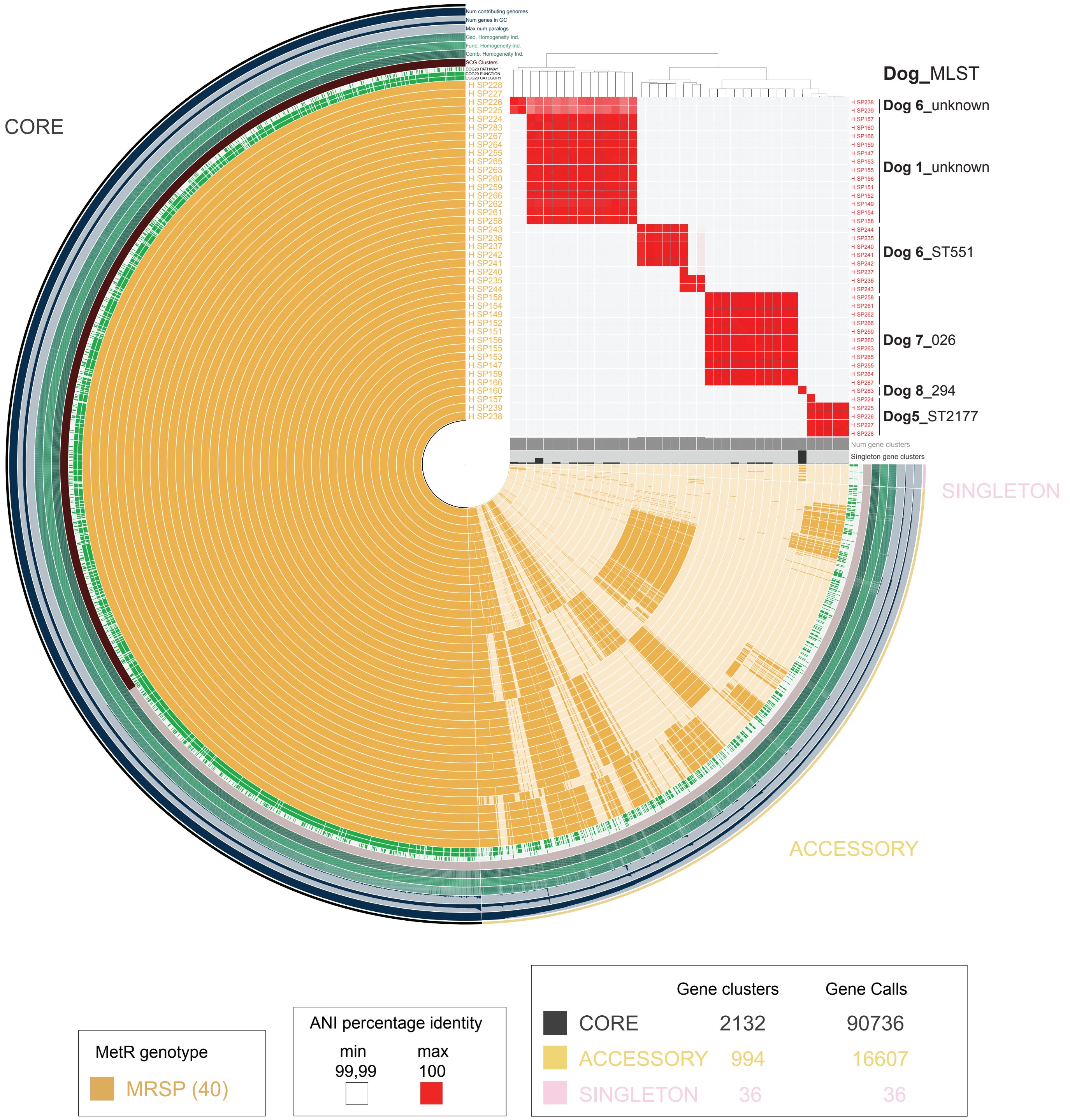

Supplementary Figure 3. Pangenome analyses of MRSP genotypes

SUPPLEMENTAL MATERIAL

Supplementary Figure 1. No significant differences were detected between *S. pseudintermedius* genomes from different body sites.

Box plots show the distribution of the (A) genome size, (B) antimicrobial resistance genes number, (C) prophages number, (D) plasmids number and (E) virulence factors number of the representative *S. pseudintermedius* isolates sequenced in this study. Boxplots shows results of *S. pseudintermedius* genomes (n=18), comparing the isolates between different body sites: inguinal n = 4, nasal n = 5, perianal n= 6, perioral n = 3. Shapiro-Wilk normality tests revealed that the data was not normally distributed. One way ANOVA & Tukey's test revealed significant differences in those cases marked by different letters, denoting significant statistical differences (p-value < 0.05).

Supplementary Figure 2. Pangenome analyses of MSSP genotypes

Pangenome results of all the *S. pseudintermedius* MSSP genomes (n=27). Core genome is by definition the part of the pangenome that is present and shared by all the genomes within the pangenome. Accessory genome is specific for a group of strains within the pangenome and Singletons are strain specific genome sequences. Visualization of pangenome analyses carried by ANVI'O. Central dendrogram clustering of samples is ordered by gene cluster presence/absence. Items order: Number of genomes gene cluster has hits (D: undefined; L: undefined). Sample order: ANIb_percentage_identity.

Supplementary Figure 3. Pangenome analyses of MRSP genotypes

Pangenome results of all the *S. pseudintermedius* MRSP genomes (n=40). Core genome is by definition the part of the pangenome that is present and shared by all the genomes within the pangenome. Accessory genome is specific for a group of strains within the pangenome and Singletons are strain specific genome sequences. Visualization of pangenome analyses carried by ANVI'O. Central dendrogram clustering of samples is ordered by gene cluster presence/absence. Items order: Number of genomes gene cluster has hits (D: undefined; L: undefined). Sample order: ANIb_percentage_identity.

Supplementary Table 1. Genomic data of the 67 main contigs assembled corresponding to the chromosomal *S. pseudintermedius* genomes isolated from the skin of healthy dogs.

Supplementary Table 2. Genomic data of the 20 additional smaller contigs corresponding to plasmids from the skin of healthy dogs.

Supplementary Table 3. MLST localization in each dog and skin body site.

Supplementary Table 4. ANI percentage identity values of the 67 *S. pseudintermedius* genomes.

Supplementary Table 5. Genomic data of the 18 representative *S. pseudintermedius* genomes isolated from the skin of healthy dogs.

Supplementary Table 6. Functional enrichment statistical analysis between MRSP genotypes and MSSP genotypes within 25 representative *S. pseudintermedius* genomes.

Supplementary Table 7. Comparison between Flye 2.8.3 vs Flye 2.9 assembly results.

Supplementary Table 8. SCCmec elements identified within the 67 *S. pseudintermedius* genomes.

Supplementary Table 9. Number of SNPs that differed between the isolates and dogs.
